# *Leptospira* Infections in Domestic and Wild Animals

**DOI:** 10.3390/pathogens9070573

**Published:** 2020-07-15

**Authors:** Giovanni Cilia, Fabrizio Bertelloni, Filippo Fratini

**Affiliations:** Department of Veterinary Sciences, University of Pisa, Viale delle Piagge 2, 56124 Pisa, Italy; giovanni.cilia@vet.unipi.it (G.C.); filippo.fratini@unipi.it (F.F.)

**Keywords:** *Leptospira*, leptospirosis, pathology, zoonosis, wildlife, infectious disease, wild boar, *Sus* scrofa, African green monkey, *Chlorocebus sabeus*, vaccine, dogs, bovine, new Pomona serovars, MLST, VNTR

## Abstract

Leptospirosis is a worldwide-distributed, re-emerging zoonosis due to the large variety of wild and domestic animal species that can play the role of natural or accidental host. Currently, specific animal species play an important role as the reservoir for particular *Leptospira* serovars, although recent investigations have highlighted new host–pathogen interactions involved in *Leptospira* epidemiology. Furthermore, the constant modification of ecosystems and wildlife habitats and the constantly increasing number of animal species moving towards urban or peri-urban areas are increasing the possibility of direct or indirect contacts between wildlife and domestic animals; furthermore, the constant modification of animal leptospirosis also causes problems for human health. The studies published in this Special Issue have evidenced and confirmed the hidden role of a large variety of animal species, domestic and wild, in the leptospirosis epidemiology. They highlighted the necessity for continuous monitoring and large-scale surveillance studies to better understand this neglected and re-emerging zoonosis.

## Introduction

*Leptospira* spp. is a Gram-negative bacterium belonging to Spirochetales order. *Leptospira* species, with more than 260 antigenically distinct serovars, have been grouped into pathogenic, intermediate, and saprophytic, with different levels of pathogenicity for animals and humans [1]. Pathogenic species of *Leptospira* are causes of leptospirosis, a re-emerging and widespread zoonosis. Moreover, intermediate *Leptospira* may be potentially pathogens and are responsible for mild infection, while saprophytic ones are spread in the environment and they are nonpathogenic [2,3].

Leptospirosis occurs in tropical, subtropical, and temperate zones, representing a public health problem due to its involving of humans, domestic and wild animals, which can be maintenance or accidental hosts [4,5]. Maintenance hosts are asymptomatic renal carriers, that contribute to maintaining and sharing the infection-shedding *Leptospira* with urine in the environment. Accidental contact with *Leptospira*-infected urine represents the first cause of infection, that could produce clinical disease [1]. So, the *Leptospira* epidemiology is closely related to the maintenance host species, generally linked to a specific *Leptospira* serovar; usually Icterohaemorrhagiae and Ballum serogroups are associated with rodents [6,7,8,9], Pomona and Tarassovi serogroups with pigs and wild boar [10,11,12,13], Bratislava serogroup with horses [14,15] and Sejroe serogroup with bovines and ovine [16,17].

To better understand the epidemiology of the *Leptospira* infection in wild and domestic animals, this Special Issue aims to bring together research studies related to investigating the role of these animals in leptospirosis epidemiology, as well as new prospective for treatment and prevention. The seven studies published in this Special Issue highlighted a wide-spectrum of *Leptospira* hosts in relation to different geographic areas and they emphasized new host−pathogen interactions in common and uncommon animal species.

Bertasio et al. performed a *Leptospira* investigation on 131,660 pigs sampled from 2002 to 2017 from 4715 farms in Northern Italy. A serological positivity rate of 13.05% was determined through Microscopic Agglutination Test (MAT). Australis was the most frequently identified serogroup (77.29%), followed by Pomona (18.47%), Tarassovi (1.51%) and Icterohaemorrhagiae (1.40%). Moreover, culture isolation and RealTime PCR were performed on 347 kidneys and 470 clinical samples, respectively. Using a Multi-Locus sequence typing (MLST) and Variable-Number Tandem Repeat (VNTR) analysis, 43 samples produced identical profiles but, after 2014, three new *Leptospira interrogans* serogroup Pomona genotypes were observed. Moreover, two isolates showed new MLST profiles and an unclassified identification by monoclonal antibodies [18].

Balboni and colleagues characterized *Leptospira* isolated in dogs with confirmed symptomatic acute leptospirosis. *Leptospira* spp. DNA were detected in urine, blood, or both in samples from nine infected dogs; obtained isolates were analyzed using the MLST technique. The isolates from two dogs were successfully typed: one belonging to Sequence Type (ST) 17 and one to ST198, *Leptospira interrogans* serogroups Icterohaemorrhagiae and Australis, respectively. The study provided the first molecular analysis aimed at identifying infectious *Leptospira* directly on DNA from biological samples of dogs. The authors showed that serogroup Australis could lead to a severe clinical presentation of leptospirosis in dogs [19].

Cilia et al. carried out an investigation aimed at evaluating the prevalence of *Leptospira* with serological, bacteriological, and molecular assays in wild boar (*Sus scrofa*) hunted in Tuscany (Italy) during two hunting seasons. In total, 287 specimens of sera, kidneys, and liver were collected to perform MAT, isolation, and RealTime PCR to detect pathogenic (*lipL32* gene), intermediate (*16S rRNA* gene), and saprophytic (*23S rRNA* gene) *Leptospira*. Within the sera, 39 (13.59%) were positive to MAT, and Australis was the most represented serogroup (4.88%), followed by Pomona (4.18%), and Tarassovi (3.14%). Moreover, four *Leptospira* cultures were positive and isolates were subjected to MLST analysis; one of them was identified as *L. borgpetersenii* serovar Tarassovi, and three as *L. interrogans* serovar Bratislava. Pathogenic *Leptospira* DNA were detected in 32 wild boar kidneys (11.15%). The characterization through the amplification of the *rrs2* gene highlighted they were positive for *L. interrogans* (23 kidneys), *L. borgpetersenii* (4 kidneys), and *L. kirschneri* (1 kidney), while nine kidneys (3.14%) were positive for intermediate *Leptospira*, all belonging to *L. fainei* [20].

Gregoire et al. investigated bovine leptospirosis. During July 2014, an emerging phenomenon of increased incidence of icteric abortions associated with leptospiral infection occurred in southern Belgium. First-line serological analyses targeting cattle-adapted serovars failed at initial diagnosis. This study provided a comprehensive description of laboratory findings—at the level of necropsy, serology and molecular diagnosis—regarding icteric and non-icteric abortions (*n* = 116) recorded during this time (years 2014–2015) and associated with incidental infection by serovars such as Grippotyphosa, Australis and Icterohaemorrhagiae. Based on these tests, a diagnostic pathway is proposed for these types of infection in cattle to establish an affordable but accurate diagnosis in the future. The investigations add insight into the understanding of the pathogenesis of bovine leptospirosis associated with serovars classically described as no maintenance host [21].

Rajeev and colleagues investigated the potential asymptomatic *Leptospira* carrier status among African green monkeys (*Chlorocebus sabeus*) on the Caribbean island of Saint Kitts. Moreover, the authors analyzed if any renal pathology could be associated with *Leptospira* exposure. Of the African green monkeys tested, 48% were positive for *Leptospira* antibodies by the microscopic agglutination test. *Leptospira* DNA was detected in 4% of kidney samples tested, using a *lipl32* gene-based PCR. Microscopic renal lesions, from minimal to severe, were evidenced in 85% of the African green monkey kidneys. Most of the African green monkeys (*n* = 26) had only minimal to mild interstitial nephritis and a few (*n* = 3) had moderate to severe lesions. The presence of interstitial nephritis was not significantly associated with *Leptospira* exposure [22].

Bertasio and colleagues detected and genotyped *Leptospira* isolated from symptomatic dogs in northeast Italy between 2013 and 2019. In total, 1631 dogs were tested using RealTime PCR, and the isolates from 193 dogs were subjected to MLST and a VNTR analysis. The bacteria were successfully isolated from 15 symptomatic dogs. Six distinct STs were found for 135 isolates, with 3 STs characterizing *Leptospira interrogans* (ST17, ST198 and ST24), 2 STs characterizing *Leptospira kirschneri* (ST117 and ST289) and 1 ST characterizing *Leptospira borgpetersenii* (ST155), revealing the circulation of the serogroups Icterohaemorrhagiae, Australis, Sejroe and Pomona. The genomic analysis of 17 samples did not result in any additional discrimination. Genotypes were compared with those of strains present in the historical internal database, and possible transmission chains were identified from rat, mouse, hedgehog, and pig. The work highlights the importance of molecular methods in revealing and identifying circulating *Leptospira* strains, and it also encourages the evaluation of the ability of commercially available vaccines to reduce the disease burden among dogs [23].

Zakharova et al. assessed the potential risk expositing to the infection as a function of environmental determinants in the Republic of Sakha (Yakutia), Russian Federation. The authors applied environmental niche modeling using leptospirosis cases in livestock and wild animals in the period from 1995 to 2019 with regard to a set of landscape, climatic, and socioeconomic variables, both for the current climate and for the projected climate for 2041–2060. The MaxEnt model performed well (AUC = 0.930), with the mean temperature of the warmest quarter, mean diurnal range, land cover type, and altitude being the most contributing variables. Consequent zoning based on the proportion of high-risk cells within each administrative unit suggested that five out of the 36 districts of the Republic are at high risk in the current climate conditions, with three more districts expected to demonstrate a high risk by 2060. The study presents the first ever attempt at leptospirosis ecological modeling in Russia. Its results correspond well to the findings of other authors and underline the importance of considering ecological factors when conducting a leptospirosis risk assessment [24].
